# Sequence and Analysis of the Genome of the Pathogenic Yeast *Candida orthopsilosis*

**DOI:** 10.1371/journal.pone.0035750

**Published:** 2012-04-26

**Authors:** Alessandro Riccombeni, Genevieve Vidanes, Estelle Proux-Wéra, Kenneth H. Wolfe, Geraldine Butler

**Affiliations:** 1 School of Biomolecular and Biomedical Science, Conway Institute, University College Dublin, Belfield, Dublin, Ireland; 2 Smurfit Institute of Genetics, Trinity College Dublin, Dublin, Ireland; Institute for Genome Sciences, University of Maryland School of Medicine, United States of America

## Abstract

*Candida orthopsilosis* is closely related to the fungal pathogen *Candida parapsilosis.* However, whereas *C. parapsilosis* is a major cause of disease in immunosuppressed individuals and in premature neonates, *C. orthopsilosis* is more rarely associated with infection. We sequenced the *C. orthopsilosis* genome to facilitate the identification of genes associated with virulence. Here, we report the *de novo* assembly and annotation of the genome of a Type 2 isolate of *C. orthopsilosis*. The sequence was obtained by combining data from next generation sequencing (454 Life Sciences and Illumina) with paired-end Sanger reads from a fosmid library. The final assembly contains 12.6 Mb on 8 chromosomes. The genome was annotated using an automated pipeline based on comparative analysis of genomes of *Candida* species, together with manual identification of introns. We identified 5700 protein-coding genes in *C. orthopsilosis*, of which 5570 have an ortholog in *C. parapsilosis.* The time of divergence between *C. orthopsilosis* and *C. parapsilosis* is estimated to be twice as great as that between *Candida albicans* and *Candida dubliniensis*. There has been an expansion of the Hyr/Iff family of cell wall genes and the JEN family of monocarboxylic transporters in *C. parapsilosis* relative to *C. orthopsilosis*. We identified one gene from a Maltose/Galactoside O-acetyltransferase family that originated by horizontal gene transfer from a bacterium to the common ancestor of *C. orthopsilosis* and *C. parapsilosis*. We report that *TFB3*, a component of the general transcription factor TFIIH, undergoes alternative splicing by intron retention in multiple *Candida* species. We also show that an intein in the vacuolar ATPase gene *VMA1* is present in *C. orthopsilosis* but not *C. parapsilosis*, and has a patchy distribution in *Candida* species. Our results suggest that the difference in virulence between *C. parapsilosis* and *C. orthopsilosis* may be associated with expansion of gene families.

## Introduction

*Candida parapsilosis* is one of the most common causes of *Candida* infection and is second only to *Candida albicans*, particularly in South America [1]. Infection rates are particularly high in premature neonates and in young children [2]. *C. parapsilosis* is found on the hands of health-care workers [3] and has been associated with major outbreaks of infection, particularly in neonatal intensive care units [4], [5], [6], [7]. The presence of central venous catheters (CVCs) and receiving parenteral nutrition are also major risk factors [8], [9].

Until recently, *C. parapsilosis* isolates (*C. parapsilosis* sensu lato) were characterized as belonging to one of three groups (I to III) [10], [11]. In 2005, Tavanti et al [12] proposed that the degree of diversity observed supported the designation of each group as a separate species; Group I remained as *C. parapsilosis* sensu stricto and Groups II and III isolates were renamed *C. orthopsilosis* and *C. metapsilosis*. Although *C. parapsilosis* sensu stricto isolates are very similar at the genetic level [13], [14], there is considerable variation among other isolates [15], [16], [17]. All species in the *C. parapsilosis sensu lato* group are members of the CTG clade, which translate CTG as serine rather than leucine [18]. Molecular analysis of the *C. parapsilosis* sensu lato species is restricted by the lack of molecular data. Whereas the *C. parapsilosis* sensu stricto genome was sequenced in 2009 [19], leading to the generation of microarrays and their application to whole genome transcriptional profiling [20], [21], [22], there is very little information available from the other species.

In recent years, several studies have measured the prevalence of *C. orthopsilosis* and *C. metapsilosis* in human infection, in particular to determine the fraction that were previously identified as *C. parapsilosis* [23], [24], [25], [26], [27], [28], [29], [30]. The estimates vary, from frequencies of 1.7% (*C. metapsilosis*) and 1.4% (*C. orthopsilosis*) in Spain [25] to 10.9% and 23.8% for *C. orthopsilosis* in South America [24]and Malaysia [26]. There is not yet sufficient information to determine whether the geographical variation reflect a difference in virulence of the pathogen or in the host response, but many of the studies are based on very small numbers and so are subject to statistical anomalies. *C. orthopsilosis* is more frequently identified than *C. metapsilosis* [31]; in addition, a very small number of cases (0.8%) originally attributed to *C. parapsilosis* are caused by a more distant relative, *Lodderomyces elongisporus* [32]. The lower numbers of infection associated with *C. orthopsilosis* and *C. metapsilosis* suggest that these species are less virulent than *C. parapsilosis*. *C. orthopsilosis* however may still be a significant cause of disease, and has been associated with two outbreaks of infection in hospitals in Texas [11] and Brazil [33].

There are few differences in drug susceptibility among the three species. *C. orthopsilosis* and *C. metapsilosis* tend to exhibit lower MICs to amphotericin B and echinocandins than *C. parapsilosis* [24], [34]. All three sensu lato species are less susceptible to echinocandins than other *Candida* species, because of a proline-to-alanine substitution in the target protein Fks1 (beta-glucan synthase) [35]. Isolates of *C. orthopsilosis* and *C. metapsilosis* have an additional isoleucine-to-valine substitution in the hot spot 2 region of Fks1 which is not associated with drug susceptibility [28]. *C. parapsilosis* sensu lato species are generally susceptible to fluconazole [24]. However, the numbers of isolates tested remain small, and there some differences have been found. For example, three resistant *C. metapsilosis* isolates were identified in Taiwan [28].

Little is known about the virulence characteristics of *C. orthopsilosis* and *C. metapsilosis*. Like *C. parapsilosis*, *C. orthopsilosis* isolates are capable of inflicting damage on reconstituted human epithelial and epidermal tissues, whereas *C. metapsilosis* is less effective [36]. *C. metapsilosis* isolates are also less efficiently phagocytosed than the other sensu lato species, and are more susceptible to host responses [37]. Growth as biofilms is a major virulence characteristic of *C. parapsilosis*. There are some reports that suggest that *C. orthopsilosis* and *C. metapsilosis* are unable to form any biofilms [23], [38], [39], whereas others show that all three species form biofilms with similar structure [40], but *C. orthopsilosis* and *C. metapsilosis* biofilms may be smaller [41].

It is highly likely that the definition of species from with the *C. parapsilosis* sensu lato group is not yet complete. For example, sequencing of the ITS region of 58 isolates from Brazil and India indicated that they fell into 4 groups, with Group IV being most closely related to Group II (*C. orthopsilosis*) [15]. We sequenced the ITS of 13 isolates identified as *C. orthopsilosis*, and showed that the majority fall into two groups, with one intermediate isolate [17]. This division was supported by an analysis of the structure of the mating type locus, which also places *C. orthopsilosis* isolates in two groups (called Type 1 and Type 2) [17]. The similarity of the regulatory proteins in the mating idiomorphs from the two types ranges from 80 to 93% [17]. The *C. orthopsilosis* type strain ATCC96139T most closely resembles the Type 1 isolates. However, the difference between the ITS sequences of the two groups is very small (3 bp in a 412 bp region) and the two Types (or sub-species) have not been experimentally or clinically distinguished. Here, we describe the genome sequence of *C. orthopsilosis* 90–125, which is characterized as a Type 2 sub-species by analysis of its mating locus, and as an intermediate species by ITS sequencing [12], [17]. There has been significant rearrangement in the chromosome structure relative to *C. parapsilosis*. We also show that some gene families associated with pathogenesis in *Candida* species are expanded in *C. parapsilosis* relative to *C. orthopsilosis*.

## Results and Discussion

### *De novo* assembly of the *C. orthopsilosis* genome using 454 and Illumina technology

We obtained the genome sequence of *C. orthopsilosis* isolate 90–125 [12] by using a combination of sequencing technologies. First, 10× sequence coverage was generated using a Roche GS FLX instrument (LGC Genomics), and assembled into 39 scaffolds using Newbler [42]. Data from a fosmid library (4800 paired-end Sanger reads) were used to verify and merge the scaffolds into 8 superscaffolds. We then overlaid Illumina data from the same strain (105× coverage from single reads) onto this structure using a guided assembly method from Velvet [43]. Our final *C. orthopsilosis* genome sequence consists mostly of sequence generated by Illumina technology, imposed on 8 chromosomes whose large-scale structure is derived from the 454 paired-end and Sanger fosmid-end data. This hybrid assembly procedure exploits the best features of the different technologies; fewer indel (insertion/deletion) errors from the Illumina data, and better contiguity from the longer Roche reads and their associated read-pair information.

The assembly consists of 8 superscaffolds, which are structurally equivalent to chromosomes. The total length is 12.6 Mb, with the largest chromosome being 2.9 Mb and the shortest 613 kb. 246 gaps remain, ranging in estimated size from 11 bp to 3 kb and likely due to repetitive sequences in the genome.

**Figure 1:**
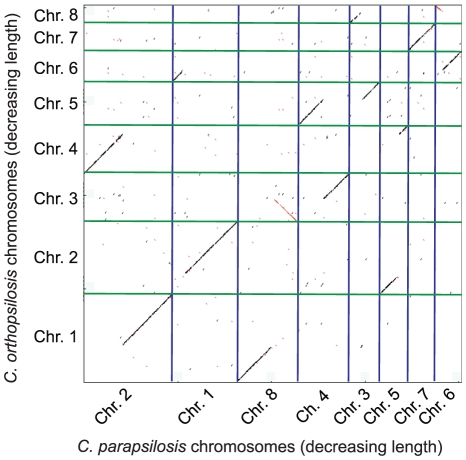
Dot matrix comparison of the *C. parapsilosis* and *C. orthopsilosis* genomes. The horizontal axis represents a joining of the 8 largest *C. parapsilosis* superscaffolds, sorted by decreasing length. The vertical axis represents a joining of the 8 *C. orthopsilosis* superscaffolds, sorted and named by decreasing length. Sequence aligning between the two species is represented in black if in the same direction, and in red if in the opposite direction.

Most *C. orthopsilosis* chromosomes show at least one translocation event when compared to *C. parapsilosis* (Figure 1). By manually identifying the major diagonals in Figure 1 and using GRIMM [44], we found that the two genomes differ by a minimum of 7 reciprocal translocation steps. In addition, we found a total of 397 inversion events between the two genomes, including 240 small inversions at single gene level [45] and 157 larger cases involving more than one gene.

### Annotation of the *C. orthopsilosis* genome

To annotate protein-coding genes we used a novel pipeline that utilizes information on conserved synteny to make ortholog inferences, which was developed for annotating genomes from the family Saccharomycetaceae [46]. The pipeline used gene sequences and gene order information from 11 *Candida* genomes contained in the Candida Gene Order Browser (CGOB) database [47] (Maguire et al, in preparation) as input. In essence, the pipeline simultaneously constructs maps of the gene locations in the newly sequenced genome and its synteny relationship to other genomes in the database.

The pipeline predicted more than 5200 gene models; some short unconserved open reading frames were subsequently removed during manual editing. Genes containing introns within the coding sequence were identified by comparison with 355 orthologs from *C. parapsilosis* [48]. We identified 387 introns in 355 *C. orthopsilosis* genes (Table S1). We identified four introns with presence/absence differences between *C. orthopsilosis* and *C. parapsilosis*; two of these are found in *C.albicans* and *C. parapsilosis* and are therefore likely to be ancestral, but have been lost in *C. orthopsilosis* (Figure S1A–B). A third intron that is present in the *C. orthopsilosis* gene *CORT0D03940* and in *C. albicans* appears to have been lost twice in parallel: once in *C. parapsilosis* and once in *L. elongisporus* (Figure S1C). The fourth intron is found only in *C. parapsilosis* (ortholog of *CORT0H01800*; Figure S1D) but exon 1 is only 3 bp long and the alignment in this region is poor, so it is difficult to define this event as a definite case of intron gain. For five other *C. parapsilosis* intron-containing genes we were unable to establish the structure of the *C. orthopsilosis* ortholog because it was located at a gap in the genome sequence.

We annotated 82 tRNA genes using tRNAscan-SE [49]. While the number of tRNAs we predicted is lower than in other *Candida* species [19], this is consistent with the 91 predictions found in *C. parapsilosis* using the same method [48]. The final version of the *C. orthopsilosis* annotation includes 5700 ORFs (including 28 pseudogenes and 65 incomplete gene models), four rRNA genes in a consensus rDNA unit, and 82 tRNA genes. 5570 ORFs have homologs in *C. parapsilosis*.

### Phylogenetic relationships and evolutionary divergence in the *C. parapsilosis* species group

**Figure 2:**
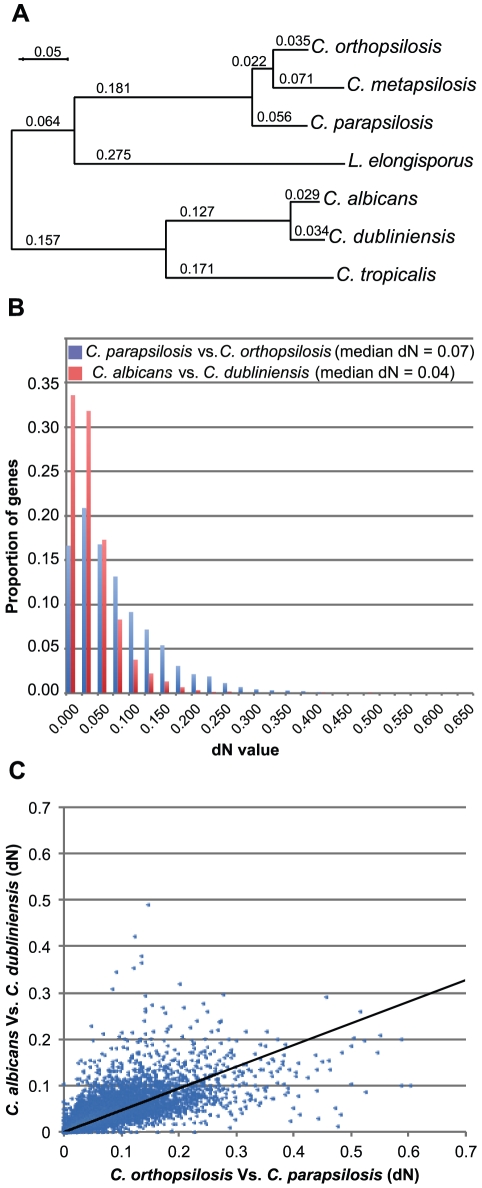
Phylogeny and sequence divergence in the *C. orthopsilosis* clade. (**A**) Phylogenetic relationship among 7 species, from maximum likelihood analysis of concatenated partial sequences of 1334 proteins. Numbers indicate branch lengths. Bootstrap values on all branches are 100%. (**B**) Histogram of distributions of nonsynonymous substitution levels (*dN*) in 5091 orthologous genes for two independent interspecies comparisons (*C. orthopsilosis* vs. *C. parapsilosis*, and *C. albicans* vs. *C. dubliniensis*). (**C**) Scatter plot showing the correlation of *dN* values for individual genes in the same two comparisons. The regression line has been forced through the origin.

To investigate the phylogenetic relationship among *C. orthopsilosis*, *C. parapsilosis* and *C. metapsilosis*, we analyzed the concatenated partial sequences of 1334 genes from all three species (see Methods). *C. orthopsilosis* and *C. metapsilosis* form a clade, with *C. parapsilosis* falling outside (Figure 2A). This topology agrees with that found by Tavanti et al [12] by analysis of one gene (the ITS1 region of rDNA), but disagrees with Kosa et al [50] whose analysis of seven genes from the mitochondrial genome placed *C. metapsilosis* outside *C. orthopsilosis* and *C. parapsilosis*. Using the Shimodaira-Hasegawa test [51] we found that the topology in Figure 2A has a significantly higher likelihood than alternatives in which either *C. orthopsilosis* or *C. metapsilosis* was an outgroup to the other two (*P*<10^−3^ in both cases).

To investigate rates of protein sequence evolution, and to compare the *C. orthopsilosis/C. parapsilosis* species pair to the *C. albicans/C. dubliniensis* pair [52], we measured the extents of nonsynonymous sequence divergence (*dN*) in 5091 orthologous genes that are present in all four species (Figure 2B). The average *dN* value for the *C. parapsilosis/C. orthopsilosis* comparison (0.70) is approximately twice that of the *C. albicans/C. dubliniensis* comparison (0.36) suggesting that divergence time between *C. parapsilosis* and *C. orthopsilosis* is twice as old as that between *C. albicans* and *C. dubliniensis*. This result is consistent with the 1.8-fold difference in branch lengths seen in Figure 2A.

Figure 2C compares the *dN* values for individual proteins in the two interspecies pairs. As expected, the rate of sequence evolution in the two comparisons is correlated for most proteins. We were interested in identifying proteins that are exceptions to this pattern, *i.e.* proteins that are evolving either unexpectedly quickly or unexpectedly slowly in the *C. orthopsilosis/C. parapsilosis* comparison relative to the *C. albicans/C. dubliniensis* comparison. Among 15 outlying genes selected from Fig. 2C that have fast rates in the *C. albicans/C. dubliniensis* pair, three code for transcription factors (Table S2). One of these is *WOR2*, a major regulator of the white/opaque switch. This switch, which is a morphological change associated with virulence [53], [54], has been described only in *C. albicans* and *C. dubliniensis.* Although *C. parapsilosis* and *C. orthopsilosis* contain orthologs of *WOR2*, they do not undergo a similar morphological switch. A second fast-evolving transcription factor in the *C. albicans/C. dubliniensis* branch is *CTA26*, which is a regulator of filamentous growth and a member of the *TLO2* family (van het Hoog, 2007). Whereas *C. albicans* and *C. dubliniensis* are the only *Candida* species known to undergo true filamentation, *C. dubliniensis* is much less efficient, and produces fewer filaments [55], [56]. The Tlo2 family has undergone a species-specific amplification in *C. albicans*, and is represented by only two members in *C. dubliniensis* [52]. *CTA26* represents the ancestral locus of the pre-amplification *TLO2* family, and is conserved in all *Candida* species. Our analysis suggests that the rapid divergence of Wor2 and Cta26 between *C. albicans* and *C. dubliniensis*, relative to the slow rate seen between *C. orthopsilosis* and *C. parapsilosis*, may be related to the increased virulence of *C. albicans.* Many of the outlier genes with relatively fast rates in the *C. orthopsilosis/C. parapsilosis* comparison are of unknown function (Table S2). Two are orthologs of genes regulated by Hap43 in *C. albicans* and may therefore be expressed in response to iron levels [57] one is a potential transcription factor of unknown function (CORT0A07620), and one is a putative cyclin-like protein.

### Gene content analysis in *C. orthopsilosis* shows little difference in singleton genes but substantial difference in gene family composition with respect to *C. parapsilosis*

Comparing the genomes of the highly pathogenic species *C. albicans* with that of its much less virulent relative *C. dubliniensis* revealed extensive gene loss and pseudogenisation in the latter species, suggesting that *C. dubliniensis* is a defective pathogen that degenerated from a virulent ancestor [52]. To determine if this is a conserved feature of pathogenic *Candida* species, we compared the gene content of *C. orthopsilosis* with that of other *Candida* species. We used CGOB to identify genes that are missing from *C. orthopsilosis*, but are present in at least four other *Candida* species (Table S3). Many apparent individual losses in *C. orthopsilosis* coincide with gaps in the genome sequence, and it is not possible to be completely sure of their absence. However, *C. orthopsilosis* is missing orthologs of *C. parapsilosis GDX1* (gentisate dioxygenase) and *FPH1* (fumarylpyruvate dehydrolase), which may explain why only *C. parapsilosis* can utilise gentisate [58].

Wohlbach et al [59] recently described the genome sequences of two members of the CTG clade, *Spathaspora passalidarum* and *Candida tenuis.* These species, together with *P. stipitis*, can naturally ferment pentose sugars such as xylose, and have therefore attracted considerable interest from the biofuel industry [60]. Although the ability to ferment xylose is rare, most of the CTG clade species (with the exception of *L. elongisporus*) can grow on xylose as a sole carbon source. Wohlbach et al [59] associated xylose assimilation with 43 genes absent from xylose non-grower species, and in particular 15 genes that are absent from *L. elongisporus* but present in all other xylose assimilators. They concluded that these genes are likely to be important for xylose assimilation, and that they were lost in the *L. elongisporus* lineage. The analysis did not include *C. parapsilosis* or *C. orthopsilosis*, the closest relatives of *L. elongisporsus* that retain the ability to grow on xylose [61]. We therefore expanded the analysis of the xylose-associated genes to include all members of the CTG clade with sequenced genomes (Figure S2). Firstly, we noticed that several genes presumed to be absent from some species are in fact present but are not annotated in the relevant genomes; many of these genes are very short (Figure S2). Secondly, 11 of the 15 genes inferred to be associated with xylose assimilation are also missing from the genomes of *C. parapsilosis* and *C. orthopsilosis*, species that retain the ability to grow on xylose. There are only 5 genes uniquely absent from *L. elongisporus* (one of which was not previously identified by Wohlbach et al [59]). Most of these encode proteins of unknown function, although one is predicted to encode an ammonium permease, and one has a potential DNA binding domain. It would be interesting to test if introducing this small number of genes into *L. elongisporus* would restore the ability to grow on xylose. Our analysis suggests that only two genes in the group are specific to the xylose fermenters (*Schefferomyces stipitis, Sp. passalidarum, C. tenuis*). One is predicted to encode a very short protein (73 aa) and one encodes a putative alpha-glucuronidase, with a conserved domain associated with the removal of alpha-1,2 linked 4-O-methyl glucuronic acid from xylans. The latter gene in particular may be an important requirement when attempting to engineer other fungi to ferment xylans. A third putative xylose specific gene identified by Wohlbach et al [59], encodes a putative saccharolysin/oligopeptidase that is a member of a family present in almost all the CTG clade species. The *Sch. stipitis* genome contains an additional copy.

Overall, our analysis indicates that whereas there is very little difference in the content of singleton genes (*i.e.* those not belonging to gene families) in *C. orthopsilosis* and other species, there are some differences in gene family composition (Table S3). Butler et al [19] found that several gene families are amplified in the genomes of pathogenic *Candida* species when compared to non-pathogenic species and to other members of the Saccharomycotina. One such amplification is the Hyr/Iff family, some of which are associated with cell wall assembly [62], [63], [64]. Our original analysis [19] identified 17 members of the Hyr/Iff family in *C. parapsilosis*. These are located in tandem arrays (ranging from two to five copies) at six genomic locations. In *C. orthopsilosis* however, there are only two sites containing Hyr/Iff proteins, both syntenic with Iff proteins in *C. parapsilosis*. We cannot determine exactly how many family members are present in *C. orthopsilosis* because they coincide with gaps in the genome sequence. However, most of the family members present in *C. parapsilosis* are absent from the equivalent positions in *C. orthopsilosis*, and there is no evidence that they have been relocated to other parts of they genome.

Many of the Hyr/Iff family members in *Candida* species are long proteins (>1,400 amino acids) that include regions of intragenic repeats (ITRs). They are predicted to contain a GPI anchor and are likely to be heavily glycosylated. They also contain secretion signals at the N termini. It is therefore assumed that they are located in the cell wall [62]. Six of the *C. parapsilosis* proteins are large and fit this general pattern, with long intragenic repeats. Two are adjacent on chromosome 6, and four are tandemly amplified on chromosome 8. At the *C. orthopsilosis* equivalent of the chromosome 8 locus, there are at least 3 Hyr/Iff genes. This locus corresponds to the location of *IFF6* in *C. albicans* and similar genes in *C. dubliniensis* and *C. tropicalis*, which may reflect the ancestral location. *C. orthopsilosis* has no Hyr/Iff genes at the counterpart of the chromosome 6 locus.

**Figure 3:**
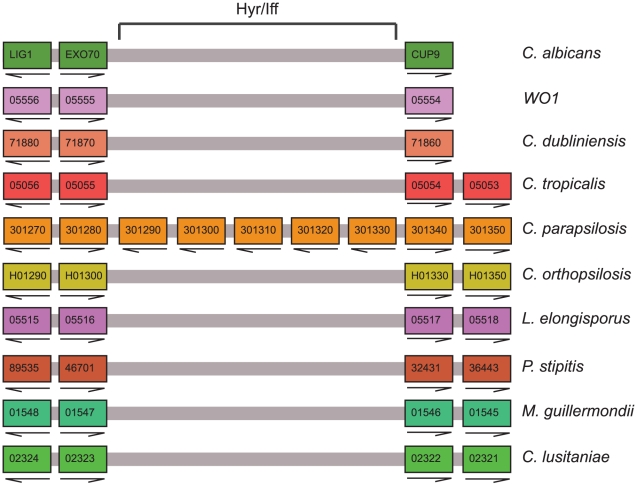
Expansion of a Hyr/Iff gene cluster in *C. parapsilosis*. The diagram is redrawn from CGOB, and represents the gene order from 11 genomes of 10 species in the *Candida* clade. Horizontal blocks of color indicate chromosomes in individual species, and pillars contain orthologs. Adjacent genes are joined by gray lines. The arrows indicate the direction of transcription. Genes 301290–301330 represent a tandem amplification of 5 Hyr/Iff genes that is unique to *C. parapsilosis.*

Most (11) of the *C. parapsilosis* Hyr/Iff family are much shorter proteins (approximately 400 amino acids). They contain the conserved N terminal domain and a secretion sequence, but lack the intragenic repeats and a GPI anchor. In contrast, only two of the 12 *C. albicans* Hyr/Iff proteins lack GPI anchors [19]. Bates et al [64] showed that deleting one of these (*IFF11*) results in a cell wall defect, and that the deletion strains have highly attenuated virulence in mouse models of infection. Iff11 is O-glycosylated and secreted [64]. The *C. parapsilosis* short Iff proteins are located in tandem arrays at four locations, on chromosome 1 (2 copies), chromosome 3 (5 copies) chromosome 4 (2 copies) and chromosome 7 (1 gene and a pseudogene). Only one of these proteins (CPAR2_301290) has a predicted GPI anchor [65]. The gene amplification is specific to *C. parapsilosis*; there are only 5 copies in *L. elongisporus*. In *C. orthopsilosis*, there are no Hyr/Iff genes at the counterparts of the loci on chromosomes 1, 3, and 7 (Figure 3 shows chromosome 3). At the chromosome 4 locus, *C. orthopsilosis* contains at least one short IFF gene (*CORT0C03535*). It is likely that amplification of the short Iff genes is important for the increased virulence of *C. parapsilosis* relative to *C. orthopsilosis*. Similarly, the loss of one Iff family member (Hyr1) has been associated with decreased virulence of *C. dubliniensis* [52].

Another virulence-related family, ALS, which is associated with adhesion, biofilm formation iron acquisition and endocytosis [19], [66], [67], [68], [69], is more similar in size between *C. parapsilosis* and *C. orthopsilosis.* There are 5 ALS genes in *C. parapsilosis* (one on chromosome 5, and four in tandem on chromosome 4 corresponding to *C. albicans ALS6-ALS7*), and at least three in *C. orthopsilosis* (an ortholog of the chromosome 5 gene, and at least two genes in tandem at the chromosome 4 site).

The CFEM family, which contain an eight-cysteine EGF-like domain, is associated with biofilm development, and acquisition of iron from heme in *Candida* [70], [71], [72], [73]. There are five members in the *C. albicans* genome, which is expanded to seven in *C. parapsilosis* by three tandem amplifications [74]. At least two of the amplifications are also present in *C. orthopsilosis*; the third position coincides with a gap in the sequence. However, whereas the role of CFEM proteins in iron acquisition is conserved in *C. albicans* and *C. parapsilosis*, the *C. parapsilosis* family has no obvious function in biofilm growth [74]. The function of the CFEM family in *C. orthopsilosis* has not been investigated.

We also noticed some amplifications that are partly shared by *C. parapsilosis* and *C. orthopsilosis*. The *C. albicans* genome contains two homologs of a transmembrane transporter, *JEN1* and *JEN2*. *JEN1* encodes a monocarboxylic acid (lactate) transporter, similar to the function of the single *S. cerevisiae* homolog [75], and *JEN2* encodes a dicarboxylic acid transporter [75]. Expression of both genes is induced in glucose-poor media, which may be important for early stage infection of mammalian hosts [75]. Previous analysis of the Jen protein family by Lodi et al [76] suggested that Jen2 is the ancestral protein, which gave rise to Jen1 through gene duplication. *S. cerevisiae* subsequently lost Jen2. There have been some rearrangements of the Jen family in the *Candida* clade; for example, the *D. hansenii* genome encodes two copies of both *JEN1* and *JEN2*, and *C. lusitaniae* has no copy of *JEN1* but has two copies of *JEN2*. However, there has been a particularly significant expansion in the *C. parapsilosis* lineage. *JEN1* is duplicated at the original locus both *C. parapsilosis* and *C. orthopsilosis. C. parapsilosis* has six additional copies, four of which are completely absent from *C. orthopsilosis* (three in a tandem cluster *CPAR2_407290–407310*, and *CPAR2_403890*). Another *JEN1* homolog is retained in both species (*CPAR2_808330/CORT0C00840*), and a further gene is severely truncated in *C. orthopsilosis* but intact in *C. parapsilosis* (*CPAR2_407250/CORT0C06620*). Thus *C. parapsilosis* has 8 *JEN1*-like genes, *C. orthopsilosis* has 3–4, and *C. albicans* has only one.

*C. parapsilosis* also has three copies of *JEN2*, including a tandem duplication at the ancestral locus where *C. orthopsilosis* has one (*CPAR2_402040/402050*, *CORT0E02090*) and one additional copy that is a pseudogene in *C. orthopsilosis* (*CPAR2_107230*, *CORT0B08430*). The amplification of the Jen family may contribute to the virulence characteristics of *C. parapsilosis*; Vieira et al [75] suggested that the ability to metabolize mono- and di-carboxylic acids is important for metabolism of *Candida* cells engulfed by macrophages.

Most of the annotated genes that are unique to *C. orthopsilosis* are small (<200 amino acids) and lack homologs in other databases. We did not find any significant families of *C. orthopsilosis*-specific genes. We identified one family that is unique to *C. parapsilosis* (represented by *CPAR2_502600*, *CPAR2_101640, CPAR2_600970, CPAR2_805490* and a pseudogene *CPAR2_301590*), and which have no similarities to any other known proteins. However, the biological function of this family is unknown.

### Analysis of drug efflux pumps

Although some species such as *Candida krusei* are inherently resistant to antifungal drugs such as azoles, exposure to sub-inhibitory concentrations can induce resistance in others, including *C. albicans, C. tropicalis* and *C. parapsilosis* [77], [78], [79]. Acquired resistance is most commonly associated with induction of expression of the drug efflux pumps (belonging to the Major Facilitator (MFS) and ATP binding cassette (ABC) superfamilies), or with overexpression or point mutations in the target enzyme lanosterol 14-alpha-demethylase (Erg11) (reviewed in [80], [81]). In. *C. albicans* gain-of-function mutations in the transcription factors *TAC1* [82] and *MRR1* [83] result in increased expression of the efflux pumps, whereas mutations in *UPC2* are associated with increased expression of *ERG11* [84]. The Ndt80 transcription factor also regulates expression of ergosterol synthesis genes [85], but appears to play no role in azole resistance [86].

At present, there are relatively few reports describing azole resistance in *C. orthopsilosis* [27], [28], [30], [31], [39], [87]. However, analysis of the dynamics of acquired resistance suggests that there may be significant differences between the underlying mechanisms in *C. albicans* and in the *C. parapsilosis* species complex [79].In *C. parapsilosis* exposure to fluconazole, voriconazole or posaconazole results in a more rapid acquisition of resistance than in *C. albicans*, and the resistance levels are stable over at least 30 days following removal of the drugs [79]. Resistance does not involve the ABC efflux pumps [79]. Transcriptional profiling indicated that fluconazole and voriconazole may induce resistance through increased expression of *MDR1* (an MFS efflux pump), similar to *C. albicans* whereas posaconazole-induced resistance is associated with increased expression of the ergosterol pathway [22].

Our analysis indicates that the Tac1, Mrr1, Upc2 and Ndt80 transcription factors are well conserved throughout the *Candida* clade, including *C. parapsilosis* and *C. orthopsilosis* (not shown). We therefore investigated the drug efflux pumps. There are approximately 95 members of the MFS family in *C. albicans* [88]. The Drug: H+ Antiporter-1 (*DHA1*) family is the largest sub-group (22 members) and includes *MDR1* which is overexpressed in azole-resistant isolates [80], and *FLU1* which confers resistance to fluconazole [89]. We find that there has been a substantial expansion of the *FLU1/TPO2* clade in *C. parapsilosis* (8 members) and *C. orthopsilosis* (6 members) (Figure S3). There has also been an expansion of the *MDR1* group; both *C. parapsilosis* and *C. orthopsilosis* contain an additional syntenic pair of Mdr1-like proteins (Figure S3). Expression of two of the *MDR1* homologs (*CPAR2_301760* and *CPAR2_603010*) is increased in azole-resistant isolates induced by exposure to fluconazole and voriconazole [22]. In contrast, there is little change in the PDR sub-family of the ABC-type transporters associated with drug efflux [90] (not shown), and activity is not affected in azole-resistant isolates [79]. It is therefore likely that azole resistance in the *C. parapsilosis* species group is associated with increased expression of the *MDR1* family.

### Alternative splicing of *TFB3* in *Candida* species

During annotation of the *C. orthopsilosis* genome we found evidence that the gene *TFB3* may be alternatively spliced in multiple *Candida* species, potentially resulting in two different versions of the protein. *TFB3* codes for one of the nine subunits of the general transcription factor TFIIH, and is highly conserved between *S. cerevisiae* and vertebrates [91]. There is no intron in *S. cerevisiae TFB3*. However, Mitrovich et al [92] identified an intron near the 5′ end of *C. albicans TFB3*, and we have found that introns are present and spliced in *TFB3* of both *C. parapsilosis* and *C. orthopsilosis* (Figure S4).

**Figure 4:**
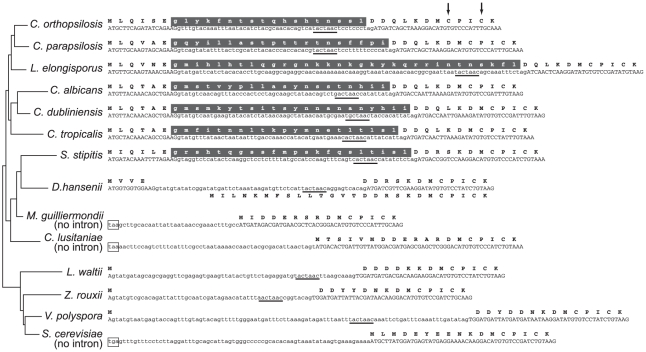
Potential in-frame translation of unspliced introns in *Candida TFB3* genes. The 5′ end of *TFB3* genes from 14 yeast species are shown, ending at a conserved region coding for the amino acid sequence DMCPICK. Exons and introns are written in upper and lower case, respectively. Gray backgrounds indicate potential in-frame translation of introns. Spliced and unspliced mRNAs have been identified in three species: *C. orthopsilosis*, *C. parapsilosis* and *C. albicans.* Probable intron branch sites are underlined. Upstream in-frame stop codons are boxed. Two possible alternative gene structures are shown for *D. hansenii*. Arrows mark two Cys residues that form part of the RING finger domain. The topology of the phylogenetic tree is from Fitzpatrick et al [126].

Further analysis revealed that the *TFB3* genes of seven species in the *Candida* clade contain introns. Remarkably, even though the introns are quite variable in length and sequence, they are all multiples of 3 nucleotides long and contain no stop codons (Figure 4). Thus unspliced mRNAs in these species could code for protein isoforms that are 18–32 amino acid residues longer than the spliced isoforms, for example extending the *C. orthopsilosis* Tfb3 protein from 343 to 361 residues. The spliced protein products from the seven species are highly similar, whereas the amino acid sequence corresponding to translation of the intron is quite variable. It is very improbable that these intron sequences are all translatable purely by chance, and more probable that the unspliced mRNA is translated under some circumstances. In analysis of published RNA-seq data from *C. parapsilosis* [48] and *C. albicans* [93], [94] and of our own unpublished data from *C. orthopsilosis*, we found that about 10% of *TFB3* transcripts retain introns in *C. orthopsilosis* and *C. albicans*, whereas about 90% of *TFB3* transcripts are unspliced in *C. parapsilosis* (Figure S4). The cause of this large variation among species is unknown.

In contrast to these seven species in the *Candida* clade that have translatable *TFB3* introns, two others (*C. lusitaniae* and *M. guilliermondii*) have no apparent intron in *TFB3*. In *Debaryomyces hansenii*, a *TFB3* intron was annotated [95], and an unspliced mRNA could potentially be translated by using a different start codon (Figure 4), but readthrough translation is not possible.

To establish whether an intron in *TFB3* was ancestrally present at the base of the *Candida* clade we re-examined *TFB3* gene structures in the family Saccharomycetaceae, which is an outgroup to this clade. Although *S. cerevisiae TFB3* has no intron, we find that many other Saccharomycetaceae species (such as *Lachancea waltii*, *Zygosaccharomyces rouxii* and *Vanderwaltozyma polyspora* in Figure 4) have an intron between the A and the TG of the start codon. This intron interrupts *TFB3* in the same phase (after base 1 of a codon) as the *Candida* intron, and the N-termini of these Saccharomycetaceae proteins are very similar, in length and in sequence, to the spliced *Candida* products. Thus a phase 1 intron appears to have been present ancestrally in the common ancestor of Saccharomycetaceae and the *Candida* clade. However, the unspliced introns in Saccharomycetaceae cannot code for extended proteins.

The amino terminus of Tfb3 contains a RING finger [96], which is a zinc-binding domain proposed to play a role in coordinating the structures of multiprotein complexes. Two zinc ions are bound by seven cysteine and one histidine residues, and two of these cysteines are located very close to the intron (arrows in Fig. 4) and conserved between yeasts and human. In fact, the N-terminus of Tfb3 from human, mouse and *Xenopus* (MDDQGCPRCK [91]) is more similar to the spliced *Candida* proteins than to *S. cerevisiae* Tfb3. Therefore, we hypothesize that the RING finger domains of the spliced and unspliced forms of Tfb3 protein may have different properties, possibly affecting the properties of the TFIIH transcription factor and hence the regulation of other genes.

### Identification of an intein sequence in *C. orthopsilosis VMA1*

**Figure 5:**
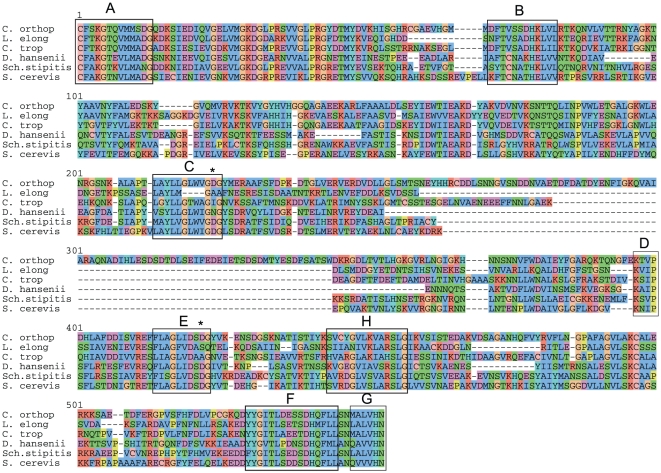
Identification of intein sequences in the Vma1 proteins of Candida species. The figure shows the alignment of the intein (VDE) sequences only; the alignment of the entire Vma1 proteins is shown in Figure S5. The motifs are labeled according to the nomenclature of Perler et al [102]. Motifs A, B, F and G are important for self-splicing. Motifs C, D, E and H are associated with homing. Two aspartic acids within the LAGLIDADG motifs in C and E that are required for homing are indicated with asterisks. The protein sequences were aligned and visualized using SeaView [122].

The *C. orthopsilosis* ortholog (*CORT0D07070*) of the *S. cerevisiae VMA1* gene includes an intein, or ‘protein intron’, whereas *C. parapsilosis VMA1* does not (Figure 5). *VMA1* codes for a vacuolar ATPase, and was the first intein-containing gene described [97], [98]. Inteins excise from the host protein, repairing the remaining ends by ligation to form a new peptide bond. The *VMA1* intein, called *VDE* or *PI-SceI* in *S. cerevisiae*, contains both self-splicing domains and a homing endonuclease domain (HEG). HEGs allow DNA coding for the intein to be copied to an empty target allele by generating a double stranded break in the DNA that is repaired by gene conversion [99].

The VDE intein is widespread in yeasts in the class Saccharomycetes, including *Candida tropicalis* [100]. We found that VDE is present in five of the 10 sequenced *Candida* genomes (*C. orthopsilosis*, *C. tropicalis*, *L. elongisporus*, *Sch. stipitis* and *D. hansenii*) and is missing in five (*C. parapsilosis*, *C. albicans*, *C. dubliniensis*, *Meyerozyma guilliermondii*, *Clavispora lusitaniae*) (Figure S5). Both groups include fully sexual and apparently asexual species [19]. In many species, inteins are inherited horizontally, because the homing mechanism ensures that they are inherited in the meiotic products [97]. However, once they are fixed in the population the HEG domain is likely to degenerate as there will no longer be a selection for function [100]. For example, Posey et al [101] showed that homing activity was functional in only two *VDE* genes from 12 species tested. The splicing domain however must remain intact so that the host protein maintains its function. This is supported by an analysis of the *Candida* inteins; the splicing domains (motifs A, B, F and G, [102]) are relatively well conserved (Figure 5).

The homing (HEG) region of inteins contains four consensus motifs, termed C, D, E and H [99], [102], [103]. Regions C and E are the LAGLIDADG motifs, which form alpha helices. Two Asp (D) residues within these motifs are essential for HEG function, both in VDE and in the *PRP8* intein in other fungi [104], [105]. The motifs are conserved in the Vma1 inteins from most of the *Candida* species, including *C. orthopsilosis*, suggesting that homing may occur (Figure 5). However, domains C and H are highly degenerate in *L. elongisporus*, and the Asp associated with domain C is substituted in this species as well as in the *C. tropicalis* and *D. hansenii* proteins (Figure 5). It is possible that the intein-containing alleles are already at a 100% frequency in populations of these species so that homing is unnecessary. It is also possible that persistence of the element is maintained by horizontal transfer [106].

### Horizontal Gene Transfer of a MAT/GAT gene

Acquisition of genes by horizontal gene transfer (HGT) can have a dramatic effect on the physiology of the recipient organism. In fungi, particularly within the Pezizomycotina, transfer of genes has changed host range, and secondary metabolism [107], [108], [109]. HGT in Ascomycetes is relatively rare, but several examples have been identified [110]. One of the best characterized examples is the acquisition of a bacterial *URA1* (dihydroorotate dehydrogenase) gene by an ancestor of *Saccharomyces cerevisiae*, which enabled the anaerobic biosynthesis of uracil [111]. Part of the biotin synthesis cluster in *S. cerevisiae* and related yeasts was built by HGT [112].

There are very few examples of HGT in *Candida* species, possibly because the reassignment of the CTG codon to serine inhibits expression of transferred genes [113]. We have previously shown that the only detectable examples of recent HGT from bacteria to this clade are restricted to the *C. parapsilosis* sensu lato species [114]. A proline racemase gene was acquired by a recent ancestor of *C. parapsilosis* sensu stricto, most likely from *Burkholderia* [114]. Similarly, a homolog of Phenazine F (PhzF) was acquired in an ancestor of *C. parapsilosis* and *C. orthopsilosis* from an alpha-proteobacteria, following the loss of the original fungal-type PhzF [114]. We confirmed that the *PhzF* gene is present in the whole genome sequence of *C. orthopsilosis* (*CORT0G03930*).

**Figure 6:**
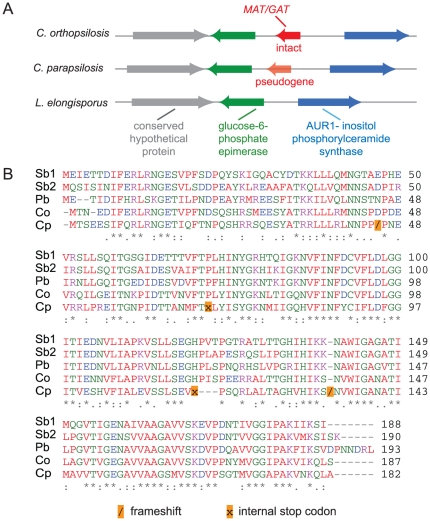
Horizontal Gene Transfer of a member of the MAT/GAT family in *C. orthopsilosis*. (**A**) Gene order surrounding the MAT/GAT gene in *C. orthopsilosis*, and the syntenic regions in *C. parapsilosis* and *L. elongisporus*. The grey, green and blue arrows represent conserved genes in all three species. The solid red arrow represents an intact ORF in *C. orthopsilosis*; the transparent red arrow represents a pseudogene in *C. parapsilosis*. (**B**) Multiple alignment of the predicted MAT/GAT proteins from *Sphingobacterium* (Sb1 = ZP_03969495.1, Sb2 = YP_004319944.1), *Pedobacter* (Pb, YP_003093425.1), *C. orthopsilosis* (Co) and *C. parapsilosis* (Cp). Yellow squares mark the presence of frameshifts (forward slash) and internal stop codons (x) that result in a pseudogene in *C. parapsilosis*.

We looked for further examples of HGT by comparing all the genes that are unique to *C. orthopsilosis* to the non-redundant database (nr) from NCBI. We also searched for *C. orthopsilosis* proteins that are more similar to bacterial proteins than to any open reading frame in *C. parapsilosis*. We identified one gene in *C. orthopsilosis* that appears to have originated by HGT from a bacterium (Fig. 6). The *C. orthopsilosis CORT0E04740* gene encodes a potential Maltose O-acetyltransferase (MAT)/Galactoside O-acetyltransferase (GAT) enzyme. Members of this family add acetyl groups to sugars in the cell wall; a well-known member is the *lacA* gene in the *E. coli* lac operon [115]. The *C. orthopsilosis* MAT/GAT is most similar to proteins from *Sphingobacterium* (68% identity), and other bacteria in the clade Bacteroidetes.

The MAT/GAT gene appears to have been gained by the common ancestor of *C. orthopsilosis* and *C. parapsilosis*. There is no MAT/GAT gene at the syntenic position in the genome of *L. elongisporus* or other *Candida* clade species. However, whereas the gene has been retained intact in *C. orthopsilosis* it has degenerated into a pseudogene in *C. parapsilosis,* which is why we did not identify it in our earlier analysis [114]. The Saccharomycotina species also contain another distantly related member of the MAT/GAT family (represented by *CORT0C03620* in *C. orthopsilosis* and *orf 19.7437* in *C. albicans*). The function is unknown, although expression of the *S. cerevisiae* ortholog *YJL218W* is increased during growth on oleate [116]. It is not known what effect the acquisition of a novel MAT/GAT enzyme has on the physiology of *C. orthopsilosis.*

### Conclusions

The availability of the *C. orthopsilosis* genome sequence makes an important contribution to comparative genome analysis of *Candida* species, and in particular to the evaluation of virulence traits. We have also provided the means for analysis of differences in biofilm formation, drug resistance and pathogenicity of the *C. parapsilosis* species group.

## Methods

### Genome sequencing and assembly

The genome sequence of the diploid *Candida orthopsilosis* strain 90–125 [12] was assembled *de novo* as a 12.7 Mb haploid consensus by LGC Genomics (www.lgc.co.uk), using a Roche GS FLX instrument with Titanium chemistry (848,000 paired reads; approximately 10× coverage). These reads were assembled into 39 scaffolds (933 contigs) using Newbler [42]. Independently, 4800 paired Sanger reads were obtained from a fosmid library from the same strain, assembled separately, and used to close some gaps in the Roche assembly. Non-repetitive fosmid read pairs were mapped to the assembly and used to verify the Roche scaffold structure and to join some scaffolds, resulting in 8 superscaffolds.

We then integrated data from 105× sequence coverage (35 million 78 bp single reads) of the same strain, obtained using an Illumina GAII at University College Dublin. Genomic DNA was extracted from cells grown in YPD at 30 C using a Genomic-tip 500/G column (Qiagen). 200 bp fragments, bound to custom adaptors, were used with the Illumina v2 Standard Cluster Generation Kit and v4 Sequencing Kits. We used the Illumina data in two ways. First, some of the remaining gaps in the genome were closed by contigs from a *de novo* Velvet assembly [43] of the Illumina data. Second, we replaced approximately 98% of the Roche data in our superscaffolds with Illumina data. This was done by using the Columbus module of the Velvet package (www.ebi.ac.uk/~zerbino/velvet), designed for resequencing projects, to make a guided assembly of the Illumina reads, using the Roche superscaffolds as a reference. We made a Python pipeline to replace parts of the Roche superscaffolds with the corresponding parts of the Illumina guided assembly, provided that the indel rate between the two regions was less than 1 indel per 2 kb. All cases where the level of indels exceeded 1/2000 were investigated manually, and most were due to poly-N regions in the Roche data.

A consensus sequence for the ribosomal DNA repeating unit was assembled manually and integrated into the scaffolds, and separately submitted to NCBI (FN812686.1). The annotated chromosomes have been submitted to EMBL with accession numbers HE681719–HE681726.

### Gene prediction and annotation

The *C. orthopsilosis* gene catalog was initially predicted using an automated annotation pipeline [117] in combination with the Candida Gene Order Browser [118], resulting in 5565 predicted ORFs. The pipeline flagged potential errors due to insertions, deletions, or frameshifts in 263 genes; where possible, these were corrected following analysis of the raw sequencing data. A Perl script was used to identify potential missing ORFs in intergenic regions, which added 200 genes to the total. All remaining models with frameshifts or internal stop codons were annotated as pseudogenes. All predictions shorter than 150 amino acids were compared to other *Candida* genomes using CGOB [118] and to the non-redundant protein database from NCBI [119]. Models with no conservation in any other species were removed from the annotation. We included 65 incomplete gene models, caused by gaps in the *C. orthopsilosis* genome, with orthologs in *C. parapsilosis*. Where the models spanned a gap in the genome sequence, only the larger of the two parts of the model was annotated. The *C. orthopsilosis* annotation was integrated into CGOB [118], which was then used as a framework for identifying orthologs of *C. orthopsilosis* genes in other *Candida* species and for identifying singletons, duplications and the insertion or deletion of genes in *C. orthopsilosis* with respect to other species. This analysis also resulted in the identification of a small number of genes that were originally overlooked in the annotation of *C. parapsilosis*, which have been added to a recent update for that species [48].

Orthologs of 355 genes containing one or more introns in the coding sequences in *C. parapsilosis* [48] were manually annotated using Artemis [120]. Introns within coding sequences in *C. orthopsilosis* were predicted by alignment of the protein sequences and manual identification of consensus splice sites. tRNA gene structures were predicted using the online version of tRNAscan-SE [49] with default parameters.

### Phylogenetic and evolutionary analyses

We used data from 4800 Sanger reads (typically ∼700 bp) from a *C. metapsilosis* fosmid library (strain ATCC96143), in combination with the genome sequences of *C. orthopsilosis* and *C. parapsilosis*, to investigate their phylogenetic relationship. We identified *C. metapsilosis* reads that had a bidirectional (BLASTX and TBLASTN) best hit relationship with a *C. orthopsilosis* gene. Reads whose translation included >1 stop codon or >2 undefined amino acid residues were discarded. We then used CGOB to extract syntenic orthologs of the *C. orthopsilosis* gene in *C. parapsilosis*, *L. elongisporus*, *C. albicans*, *C. dubliniensis*, and *C. tropicalis*. Only 1972 loci with an ortholog in all 6 species were retained. For each of these we aligned the 6 proteins with the translation of the *C. metapsilosis* read, using Clustal Omega [121]. To remove poor-quality alignments we then discarded all gapped sites, and retained each locus only where ≥50 residues were identical among all 7 species. Alignments that met these criteria were concatenated to make a superalignment containing 262,175 amino acid sites, derived from 1334 genes. A maximum likelihood phylogenetic tree was constructed from the superalignment, using PhyML as implemented in SeaView [122]. The parameters were the LG substitution model, 4 rate categories, SPR+NNI branch interchange, with 5 randomized starting trees. We verified the topology and carried out the Shimodaira-Hasegawa test using AAML from the PAML package [123].

To calculate nucleotide substitution levels, a list of 5091 genes with orthologs in *C. orthopsilosis*, *C. parapsilosis*, *C. albicans* and *C. dubliniensis* was extracted from CGOB. Pairwise alignments within each group were made with PAL2NAL [124] removing positions with gaps. CodeML [125] was used to calculate *dN* and *dS* values, using the F3X4 model, no variation among sites, and estimated values for kappa, omega and alpha. To identify genes with genes whose evolutionary rate may have changed between the *C. orthopsilosis*/*C. parapsilosis* pair and the *C. albicans*/*C. dubliniensis* pair, we first identified the 30 genes with the highest dN values for each comparison, and then sorted these genes by the ratio of divergence levels in the two species. Table S2 shows the 15 genes at each extremity.

To quantify genomic rearrangement between *C. orthopsilosis* and *C. parapsilosis*, we used GRIMM [44] to study large rearrangements identified using dot-matrix plots, and custom scripts to study small rearrangements. Genes without an ortholog in *C. parapsilosis* were ignored.

### Gene Family analysis

To investigate gene content differences between *C. orthopsilosis* and *C. parapsilosis* we used orthology assignments obtained from CGOB (Maguire at al., in preparation). We considered *C. orthopsilosis* genes that had an ortholog in at least four *Candida* species but not in *C. parapsilosis*, and *C. parapsilosis* genes with orthologs in at least four *Candida* species but not in *C. orthopsilosis*. Missing genes were verified manually. Species-specific pseudogenes and ORFs smaller than 150 amino acids were ignored. We also ignored all cases in *C. orthopsilosis* that corresponded with a gap in the genome sequence. The final list is included in Table S3.

## Supporting Information

Figure S1 **Intron gains and losses in** ***C. orthopsilosis*** **and** ***C. parapsilosis*** **.**
(TIF)

Figure S2 **Conservation of xylose assimilation genes in CTG clade species.**
(PDF)

Figure S3 **Phylogenetic analysis of the Drug H+ Antiporter-1 (** ***DHA1*** **) subfamily of the Major Facilitator Superfamily (MFS) in** ***C. orthopsilosis, C. parapsilosis*** **and** ***C. albicans*** **.**
(TIF)

Figure S4 **Examples of RNA-seq reads showing spliced and unspliced forms of the TFB3 transcript in three** ***Candida*** **species.**
(TIF)

Figure S5 **Complete multiple alignment of the VMA proteins in** ***Candida*** **species.**
(EPS)

Table S1 **Intron information for** ***C. orthopsilosis*** **.**
(XLS)

Table S2 **Genes with extreme differences in the dN rates.**
(XLS)

Table S3 **Genes missing from** ***C. orthopsilosis*** **.**
(XLS)
