# Supplementary figures and images for: Sequence and Analysis of the Genome of the Pathogenic Yeast Candida orthopsilosis
Source: PLoS One. 2012 Apr 26;7(4):e35750. doi: 10.1371/journal.pone.0035750 (PMC3338533; doi:10.1371/journal.pone.0035750)

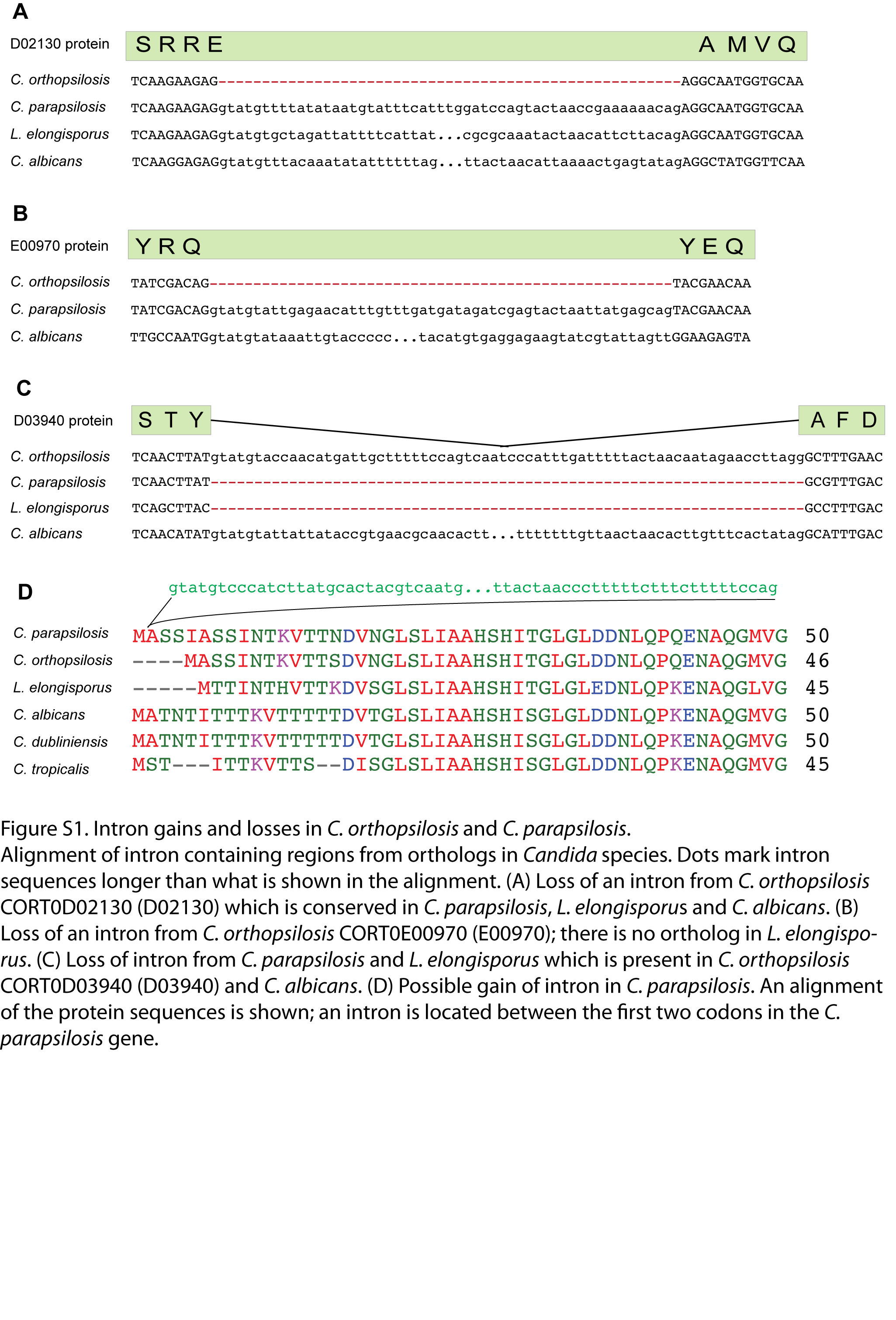

Supplement: Figure S1 — Intron gains and losses in C. orthopsilosis and C. parapsilosis . (TIF) [file pone.0035750.s001.tif]

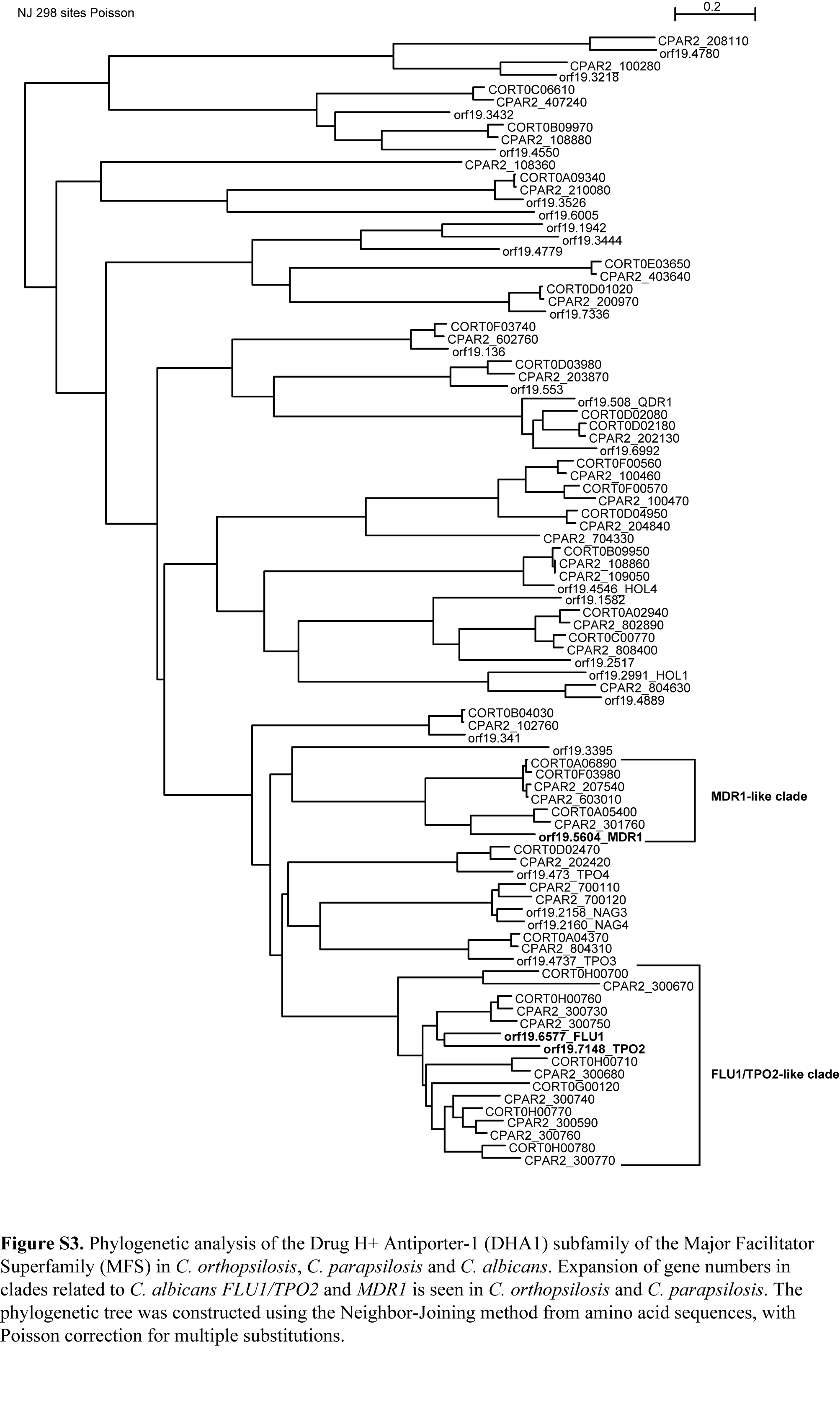

Supplement: Figure S3 — Phylogenetic analysis of the Drug H+ Antiporter-1 ( DHA1 ) subfamily of the Major Facilitator Superfamily (MFS) in C. orthopsilosis, C. parapsilosis and C. albicans . (TIF) [file pone.0035750.s003.tif]

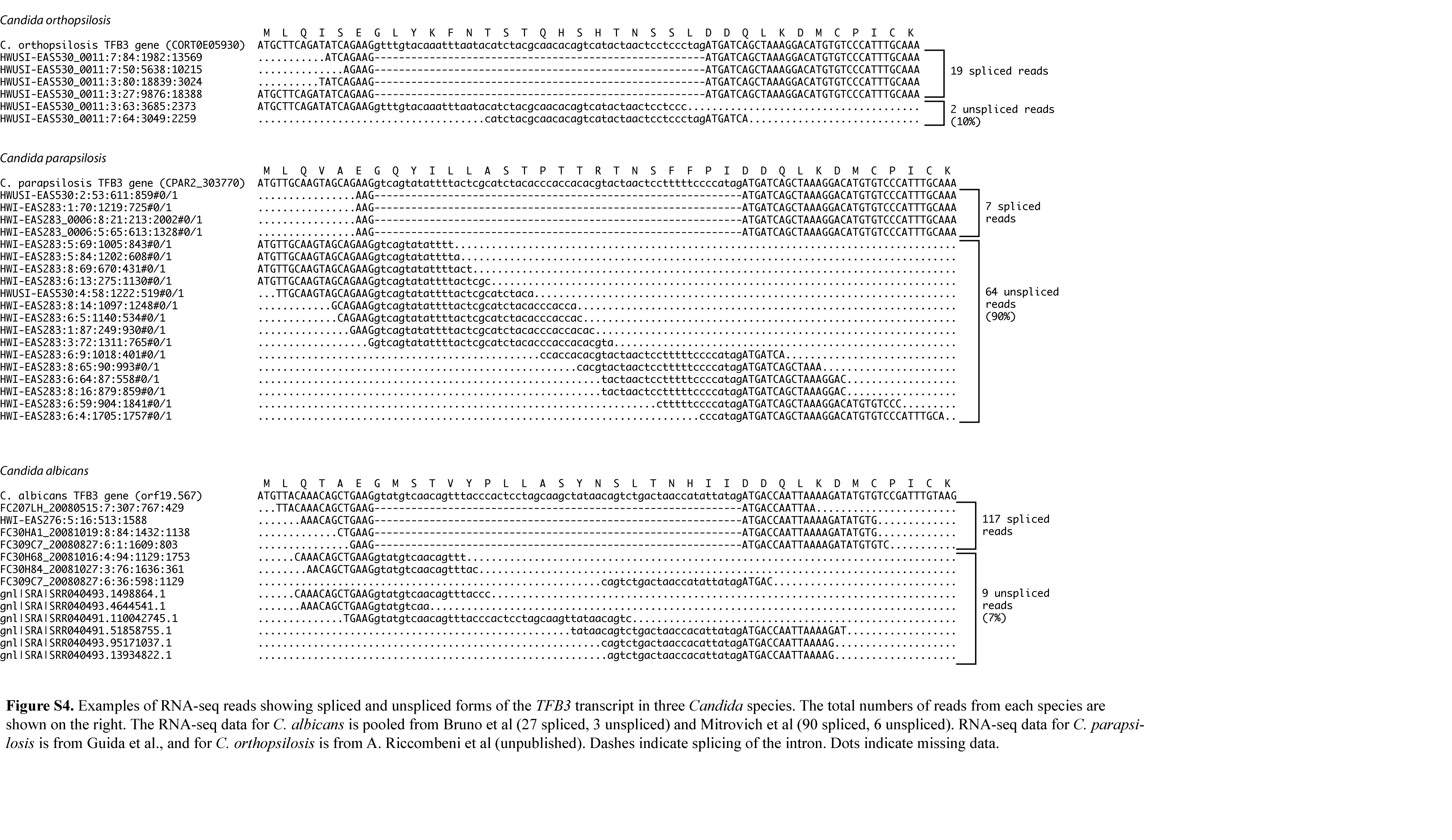

Supplement: Figure S4 — Examples of RNA-seq reads showing spliced and unspliced forms of the TFB3 transcript in three Candida species. (TIF) [file pone.0035750.s004.tif]
