# Supplementary material for: Sequence and Analysis of the Genome of the Pathogenic Yeast Candida orthopsilosis
Source: PLoS One. 2012 Apr 26;7(4):e35750. doi: 10.1371/journal.pone.0035750 (PMC3338533; doi:10.1371/journal.pone.0035750)
Supplement: Figure S2 — Conservation of xylose assimilation genes in CTG clade species. (PDF) [file pone.0035750.s002.pdf]

Figure S2. Conservation of xylose assimilation genes in CTG clade species.

C. albicans	*C. dubliniensis*	*C. tropicalis*	*C. parapsilosis*	*C. orthopsilosis*	*L. elongisporus*	*C. lusitaniae*	*M. guillermondii*	*D. hansenii*	*Sch. stipitis*	*C. tenuis*	*S. passalidarum*	**OGG***	**Predicted function**
									PICST_84532	CANTEDRAFT_110083	SPAPADRAFT_131559	724	unknown
									PICST_61283	CANTEDRAFT_133492	SPAPADRAFT_158400	2043	997 aa, alpha-glucoronidase, remove the alpha-1,2 linked 4-O-methyl glucuronic acid from xylans
									PICST_51091			2766	saccharolysin/oligopeptidase
orf19.6242									PICST_29646			3637	cytokinesis, SH3 domain, tandem duplication in Ca and Pstip.
orf19.2836	CD36_27840								PICST_57799	CANTEDRAFT_117010	SPAPADRAFT_59837	1331	similar to carbonic anhydrases
						CLUG_05744	PGUG_01260	DEHA2E18194g	PICST_37102	CANTEDRAFT_115648	SPAPADRAFT_60047	295	unknown
							PGUG_00546	DEHA2D14894g	PICST_52103	CANTEDRAFT_114218	SPAPADRAFT_57769	590	2-dehydro-3-deoxy phosphoheptonate aldolase
orf19.6137	CD36_32940						PGUG_02653	DEHA2E04422g	PICST_33357	CANTEDRAFT_134955	SPAPADRAFT_65155	3219	unknown
orf19.7457	CD36_86650	CTRG_5696					PGUG_01301	DEHA2F04378g	PICST_12953	CANTEDRAFT_112665	SPAPADRAFT_62371	4749	protein with Mob2-p dependent hyphal regulation
orf19.6413	CD36_34030	CTRG_03080					PGUG_01933	DEHA2A11616g	PICST_56697	CANTEDRAFT_100642	SPAPADRAFT_59250	70	unknown
orf19.862	CD36_18300	CTRG_01198						DEHA2E20592g	PICST_40869	CANTEDRAFT_116088	SPAPADRAFT_50369	4486	unknown
orf19.872	CD36_18230	CTRG_01196				CLUG_05388		DEHA2E20636g	PICST_66811	CANTEDRAFT_136564	SPAPADRAFT_60843	2393	unknown
						CLUG_01258	PGUG_03345	DEHA2E13046g	PICST_33418	CANTEDRAFT_135274	SPAPADRAFT_61145	3073	unknown
		CTRG_04304				CLUG_01180	PGUG_00389	DEHA2G06446g	PICST_66312	CANTEDRAFT_135458	SPAPADRAFT_57695	5534	endo-1,4-beta-glucanase
orf19.4828	CD36_09090	CTRG_03393				CLUG_03846	PGUG01712	DEHA2B10934g	PICST_5163	CANTEDRAFT_136328	SPAPADRAFT_139220	584*	Hap43-repressed
orf19.5258	CD36_11300	CTRG_03444				CLUG_02421	PGUG_03571	DEHA2C12496g	PICST_29610	CANTEDRAFT_136211	SPAPADRAFT_61805	5595*	unknown
orf19.2701	CD36_42800	CTRG_00343				CLUG_02389	PGUG02389	DEHA2G18656g	PICST_38374	CANTEDRAFT_107031	SPAPADRAFT_58628	3329*	unknown
orf19.6307	CD36_30250	CTRG_00531				CLUG_03193	PGUG_01884	DEHA2G22594g	PICST_50168	CANTEDRAFT_98619	SPAPADRAFT_148709	5433*	unknown
orf19.6024	CD36_00770	CTRG_04386				CLUG_00971	PGUG_04695	DEHA2D09812g	PICST_61958	CANTEDRAFT_112621	SPAPADRAFT_130993	342*	unknown
orf19.2283	CD36_21530	CTRG_01731				CLUG_05809	PGUG_00441	DEHA2F23584g	PICST_62322	CANTEDRAFT_104067	SPAPADRAFT_60302	3376*	3-dehydroquinate dehydratase
orf19.3285	CD36_25800	CTRG_01014				CLUG_03049	PGUG_03252.1	DEHA2E17380g	PICST_62836	CANTEDRAFT_127228	SPAPADRAFT_59967	3977*	unknown
orf19.1587	CD36_17400	CTRG_01322				CLUG_00950	PGUG_02985	DEHA2G04202g	PICST_65135	CANTEDRAFT_117185	SPAPADRAFT_60799	3804*	Glucose transporter
orf19.6899	CD36_71030	CTRG_04849				CLUG_05662	PGUG_00371	DEHA2F22044g	PICST_78306	CANTEDRAFT_113866	SPAPADRAFT_63741	2954*	Oxidoreductase
orf19.826	CD36_18630	CTRG_01446				CLUG_00154	PGUG_00714	DEHA2D11000g	PICST_83654	CANTEDRAFT_133133	SPAPADRAFT_71074	4574*	RNA Pol II co-factor
orf19.3572	CD36_19880	CTRG_01464				CLUG_00147	PGUG_00720	DEHA2D11154g	PICST_59570	CANTEDRAFT_117672	SPAPADRAFT_60486	3221*	unknown
orf19.7619	CD36_35300	CTRG_05812	cpar2_200420	CORT0D00510		CLUG_01546	PGUG_02961	DEHA2G04642g	PICST_75429	CANTEDRAFT_91521	SPAPADRAFT_59067	5585*	putative mitochondrial inner membrane protein
orf19.4446	CD36_06750	CTRG_03955	cpar2_208050	CORT0A07380		CLUG_05847	PGUG_00471	DEHA2F24442g	PICST_47780	CANTEDRAFT_93101	SPAPADRAFT_48191	1356*	similar to ammonium permeases
orf19.5841	CD36_17700	CTRG_01332	cpar2_212340	CORT0A11670		CLUG_04534	PGUG_03006	DEHA2G03784g	PICST_56294	CANTEDRAFT_128831	SPAPADRAFT_60779	1345*	unknown
orf19.5051	CD36_07330	CTRG_03979	cpar2_208330	CORT0A07620		CLUG_05881	PGUG_03132	DEHA2F26312g	PICST_32611	CANTEDRAFT_136915	SPAPADRAFT_48209	5052*	DNA binding domain
orf19.6222.1			cpar2_208910	CORT0A08180				DEHA2D14388g	PICST_37629	CANTEDRAFT_114047	SPAPADRAFT_69538	293	unknown
orf19.3655	CD36_60640	CTRG_02935	cpar2_602930	CORT0F03900		CLUG_03693	PGUG_03806		PICST_31932	CANTEDRAFT_115038	SPAPADRAFT_52433	1217	unknown
orf19.4946	CD36_12240	CTRG_03592	cpar2_801750	CORT0A01840			PGUG_03192		PICST_29111	CANTEDRAFT_135403	SPAPADRAFT_153457	4572	unknown
orf19.6027	CD36_00740	CTRG_04379	cpar2_110170	CORT0B11280				DEHA2D09724g	PICST_32723	CANTEDRAFT_133175	SPAPADRAFT_47834	131	unknown
orf19.5489	CD36_20660	CTRG_01772	cpar2_104730	CORT0B05930	LELG_01881			DEHA2F02068g	PICST_32316	CANTEDRAFT_133951	SPAPADRAFT_50010	2825	unknown
orf19.3678	CD36_02060	CTRG_04622	cpar2_106200	CORT0B07440	LELG_01642		PGUG_01149	DEHA2C02310g	PICST_30582	CANTEDRAFT_96322	SPAPADRAFT_57934	784	unknown, not necessary for viability
orf19.4332	CD36_52800	CTRG_05564	cpar2_303110	CORT0E05250	N	N	PGUG_02676	DEHA2G07964g	PICST_46903	CANTEDRAFT_92321	SPAPADRAFT_138221	1917	unknown
orf19.6474	CD36_72160	CTRG_05027	cpar2_702760	N	LELG_05090	CLUG_04177	PGUG_00209	[illegible]44g	PICST_30997		[illegible]606	2028	unknown, membrane-localized protein
orf19.4837.1			cpar2_804880	CORT0A04880				DEHA2B10604g	PICST_40454	CANTEDRAFT_111283	SPAPADRAFT_141621	2405	unknown
orf19.1367.1			cpar2_407330	CORT0C06670				DEHA2B05654g	PICST_44644	CANTEDRAFT_114646	SPAPADRAFT_135701	3442	unknown
orf19.1374	CD36_23840							DEHA2B13310g	PICST_58235	CANTEDRAFT_113310	SPAPADRAFT_136106	5073	unknown
orf19.216.1			cpar2_806510	CORT0C02640				DEHA2B13508g	PICST_59544	CANTEDRAFT_131682	SPAPADRAFT_137450	1116	unknown
orf19.4192.1	CD36_60590		cpar2_602880	CORT0F03850				DEHA2F15334g	PICST_59957	CANTEDRAFT_115034	SPAPADRAFT_157243	3365	unknown
orf19.1026.1	CD36_03580		cpar2_105090	CORT0B06290				DEHA2A09548g	PICST_62408	CANTEDRAFT_114339	spas_972	1788	unknown

43 genes identified by Wohlbach et al (2011) that are associated with fungi that grow on xylose. Annotated orthologs are shown in orange boxes. Genes that are present but are not included in the current annotations are indicated by light orange. White boxes with (N) indicate a gap in the genome sequence. Blue boxes indicate homologs are present

*OGG = orthologous gene group (from Wohlbach et al). OGGs indicated with an asterisk were suggested to be
Genes highlighted in green are common to xylose fermenters, and genes highlighted in purple are uniquely missing from L. elongisporus, which cannot metabolize xylose.
